# A multinational cross-sectional study on knowledge, attitudes, and practices towards magnesium supplements

**DOI:** 10.3389/fphar.2025.1550695

**Published:** 2025-04-28

**Authors:** Rana Abutaima, Muna Barakat, Samar Thiab, Hana M. Sawan, Malak Amer, Reem Alzayer, Doaa H. Abdelaziz, Noha O. Mansour, Fatima Saleh, Wael Abu Dayyih

**Affiliations:** ^1^ Faculty of Pharmacy, Zarqa University, Zarqa, Jordan; ^2^ Faculty of Pharmacy, Applied Science Private University, Amman, Jordan; ^3^ Clinical Pharmacy Practice, Mohammed Al-Mana College for Medical Sciences, Dammam, Saudi Arabia; ^4^ Department of Clinical Pharmacy, Faculty of Pharmacy, Al-Baha University, Al-Baha, Saudi Arabia; ^5^ Department of Clinical Pharmacy, The National Hepatology and Tropical Medicine Research Institute, Cairo, Egypt; ^6^ Clinical Pharmacy and Pharmacy Practice Department, Faculty of Pharmacy, Mansoura University, Mansoura, Egypt; ^7^ Department of Medical Laboratory Sciences, Faculty of Health Sciences, Beirut Arab University, Beirut, Lebanon; ^8^ Faculty of Pharmacy, Mutah University, Karak, Jordan

**Keywords:** magnesium salts, muscle relaxants, spasm, insomnia, magnesium supplements, over the counter, bone health, migraine

## Abstract

**Background:**

Magnesium is a vital mineral with a crucial role in different biochemical reactions. There is a contradicting evidences about its role in maintaining bone, muscle and cardiovascular health. Recently, magnesium supplements gained attention due to claimed effectiveness in improving sleep quality and relieving muscle spasm.

**Aim:**

This study aimed to assess Arab communities’ knowledge, attitudes and practices regarding magnesium supplementation.

**Methods:**

A cross-sectional self-administered survey was disseminated to collect responses from different Arab countries. Descriptive statistics were calculated for demographics. Data normality was assessed using Shapiro-Wilk test. Associations between sociodemographic variables and knowledge, attitudes and practices were explored using simple and multiple linear regression. Significance level was considered at p < 0.05.

**Results:**

A total of 1,445 responses were collected. Mean (±SD) knowledge scores were low 8.61 ± 5.9. Eighty seven percent recognized magnesium role in alleviating muscle spasm and sleep disorders (83%). Magnesium citrate was the mostly used magnesium salt (37.43%). Neutral attitudes were observed with a mean score of 3.02 ± 0.66. Only 31.8% reported using magnesium supplements, 36.1% of them received a medical consultation. Having poly cystic ovary (p = 0.033), relying on scientific articles (0.004), receiving pharmacist consultation (p = 0.019) significantly associated with higher knowledge.

**Conclusion:**

Despite the huge debate regarding magnesium benefits in maintaining muscle, bone health and improving sleep quality, this study found that there is a significant gap in knowledge and neutral attitude towards magnesium supplementation in Arab communities. These findings emphasizes the need for educational campaigns targeting the public on the rationale use of supplements.

## Introduction

The global dietary supplement market is expanding at an exponential rate. Data from the National Health and Nutrition Examination Survey (NHNES) indicates that since the 1970s, interest in it has grown (Mahdavi-Roshan et al., 2021; Lam et al., 2022). In a study conducted in 2022 in six-Arabic speaking countries, around two thirds of the participants revealed that they used supplements (Amer et al., 2024). Similarly, a similar prevalence was also reported in a study conducted in Saudi Arabia (Syed et al., 2022). In a cross-sectional study also form Saudi Arabia, magnesium was one of the supplements found to be consumed by almost 30% of the participants (Alhashem et al., 2022).

The risk of several chronic conditions, including metabolic syndrome and cardiovascular disease, as well as certain cancers (esophageal, prostate, and colon) and overall mortality, has been linked to low magnesium intakes combined with high calcium intakes and high calcium-to-magnesium intake ratios (Costello et al., 2021).

Magnesium is the fourth most abundant mineral in the human body. It is predominately found intracellularly in bone tissues and other organs and only small amounts of around 1% is found in blood (Beckstrand and Pickens, 2011).

Magnesium is an essential ion that plays an important role in supporting and sustaining human health, as it is involved in hundreds of enzymatic reactions responsible for metabolism as well as energy production and protein synthesis (De Baaij et al., 2015). It was demonstrated that magnesium is essential for various physiological functions particularly in the skeletal muscles, heart and brain (De Baaij et al., 2015; Faryadi, 2012).

Metabolism of magnesium was reported to change with aging as its absorption via the intestine gets impaired and its renal elimination increases leading to deficiency (Barbagallo et al., 2021). It was also reported that many chronic diseases were associated with magnesium deficiency, including type-2 diabetes, many cardiovascular disorders such as stroke and hypertension as well as airway constrictive syndromes, muscle diseases, bone fragility, cancer in addition to stress-related conditions (Barbagallo et al., 2021).

Magnesium supplementation was found to probably be beneficial in individuals who suffer from hypomagnesemia, elderly and alcoholics (Wang et al., 2017). A systematic review found that magnesium supplementation reduced insulin resistance in patients who also suffer from hypomagnesemia (Morais et al., 2017). In another systematic review conducted to evaluate magnesium supplementation in individuals suffering from subjective anxiety and stress found an evidence indicating suggestive benefits of magnesium supplementation in vulnerable samples (Boyle et al., 2017). Positive evidence on the benefits of magnesium intake in reducing muscle soreness, rating of perceived exertion, and improved perceived recovery was also reported by Alyssum et al. (2022). Magnesium may also have protective effects against some cardiovascular diseases, may delay the progression of chronic kidney disease (Panta and Regmi, 2024), and it was found to achieve a small but clinically significant reduction in blood pressure (Kass et al., 2012). Moreover, it was found to be effective in alleviating mild-to-moderate depression (Tarleton et al., 2017).

Magnesium supplementation is generally well tolerated (Greupner et al., 2020), however a large share of the public obtain health information from non-medical sources such as the internet, social media, family and friends (El et al., 2016; AlMuammar et al., 2021), which can lead to inappropriate self-medication and worsen pre-existing health issues, emphasizing the importance of enhanced public health education (Zaidi et al., 2023). Furthermore, sociodemographic characteristics such as age and education can have a considerable impact on knowledge and attitudes toward magnesium supplementation (Shahid et al., 2022), and people with poor knowledge might be unaware of potential drug interactions that can complicate their health management (Shahid et al., 2022).

The rationale for this study is to gather demographic information and evaluate awareness of magnesium’s uses among Arab communities. In addition, to identify gaps in understanding and behavior, thus the results would help inform public health strategies and healthcare interventions to promote safe and informed magnesium use in the Arab world.

This study aims to comprehensively evaluate Arab communities' knowledge, attitudes and practices toward magnesium use, with a focus on determining how sociodemographic variables, information sources, and personal health experiences influence views and understanding. Exploring these relationships can yield insights that will help drive public health campaigns and educational initiatives focused on raising awareness of magnesium’s health benefits and potential hazards. Such activities are critical for empowering informed decision-making among individuals and improving health outcomes within communities.

Given the increasing prevalence of dietary supplements, researching community attitudes toward magnesium is both relevant and essential. This study will expand literatures that are aiming at filling knowledge gaps and increasing health literacy in dietary supplementation, ultimately promoting better health and wellbeing in Arab communities.

### Ethical approval

Ethical approval for this study was obtained from the Institutional Review Board (IRB) committee at Applied Sciences University on 4 April 2024, following the submission of the study protocol and consent form (approval number: 2024-PH-11). Data collection took place from June 19th to 20th September 2024. Participants provided informed consent prior to responding and were assured that their data would remain confidential and anonymized, to be used merely for research purposes. They were also informed of their right to withdraw from the study at any time before submitting their responses. Any adult individual living in an Arab country was eligible to take part in the study.

## Methodology

### Questionnaire development and structure

This was a self-administered survey developed using Google^®^ Forms and distributed to the public across Arabic speaking countries (i.e. Jordan, Egypt, Saudi Arabia, Syria, Iraq, Palestine, Lebanon, Yemen, Qatar, United Arab Emirates, Bahrain, Kuwait, Sudan, Tunis, Libya, Algeria, Morocco, Comoros, Oman, Mauritania, Somalia, Djibouti) through social media platforms (i.e. Meta platforms). This method of data collection has been widely used in recent public health and cross-sectional studies due to its accessibility, scalability, and ability to reach diverse populations across different regions (El et al., 2016; AlMuammar et al., 2021; Alyami et al., 2020). The questionnaire was developed and validated through an extensive literature review on the various indications and uses of magnesium. It is structured into four main sections. The first section collects demographic information from participants, including age, gender, monthly income (US dollars), country of residence (any of the 22 Arab countries), and the presence of chronic diseases.

The second section evaluates the knowledge of Arab communities regarding magnesium use, participants were asked whether magnesium can be taken without a prescription, its indications, side effects, and the possibility of drug interactions with commonly used medications and supplements (e.g., diazepam, paracetamol, diphenhydramine hydrochloride, orphenadrine, valerian root, vitamin C) (Simental-Mendía et al., 2016; von Luckner and Riederer, 2018; John et al., 2016; Landon and Young, 1993; De Oliveira et al., 2024; Rondanelli et al., 2021; Guerrera et al., 2009; Moretti, 2021; Knox et al., 2024). Participants were provided with pictures for the supplements and medications alongside generic names to ensure public recognition of these medications and supplements. Participants were also asked about magnesium’s use in the prevention of muscle spasms and sleep disorders.

In the third section, a five-point Likert scale; strongly agree, agree, neutral, disagree, strongly disagree was used to assess the community’s awareness of magnesium, including safety, effectiveness, long-term benefits, and its role in improving bone health, diabetes, migraine, cardiovascular disease, and premenstrual symptoms.

The final section assessed participants’ practices related to magnesium use. Only those who had used magnesium in the past 12 months proceeded to this section. Participants were asked whether they consulted a physician or had their magnesium blood levels tested before use. Additionally, the dosage, dosing frequency, and perceived benefits (rated on a scale of 1–10) were examined. Questions also covered the source of magnesium supplements, methods for verifying authenticity, the occurrence of side effects, sources of information, and other co-administered supplements (e.g. Vitamin D, Omega, Iron and Folic Acid). The complete questionnaire, along with the corresponding scores, is available in the Supplementary Appendix SA1.

### Validity and reliability

The questionnaire was developed in Arabic and then translated into English by two bilingual academics who are fluent in both languages. The Arabic version of the questionnaire was distributed, and it is the official language of the target group countries. The questionnaire method was adopted as it is a widely recognized and efficient approach for assessing knowledge, attitudes, and practices in public health research (Launiala, 2009). It allows for standardized and scalable data collection across diverse and geographically dispersed populations. The original questionnaire was developed in Arabic, the official language of the targeted Arab countries, to ensure clarity, accessibility, and accurate comprehension of the content by participants from various educational backgrounds (Sousa and Rojjanasrirat, 2011; Tsang et al., 2017). Administering the survey in the native language improved response reliability and minimized the risk of misinterpretation.

Face and content validity were established through a pilot test, involving an equal sample of both the general public (n = 5) and academics (n = 5) with expertise in the field. Pilot participants were asked to provide feedback on any scientific terminology, jargon or ambiguous questions. Additionally, the academics were asked to review the structure and content to ensure comprehensive coverage of all relevant topics. Examples of the feedback received include separating questions on sleep disorders and muscle spasms into two discrete items, adding other indications for magnesium use, and providing examples of magnesium brands that participants might have used.

The decision to adopt a self-administered survey format was based on several key considerations. First, the study was conducted across multiple Arab countries, and an online self-administered format allowed for broader geographic reach and easier access to a diverse population. Second, it ensured participant anonymity, which is crucial when assessing personal health behaviors and supplement use, and likely reduced social desirability bias. Additionally, the online format was practical and cost-effective, enabling efficient data collection from a large sample while maintaining participant convenience. Lastly, since the study focused on self-reported knowledge, attitudes, and practices, a self-administered design was deemed appropriate and consistent with established methodologies in similar multinational KAP studies.

### Sample size calculation

Considering the total Arab population, estimated at 473.27 million, a minimum sample size of 385 was calculated using an online calculator (Raosoft, 2004). This was based on a 0.05 margin of error, a 95% confidence interval (CI), and a response distribution of 50%.

### Scoring

Calculation of the knowledge scores was based on participants’ responses, where each correct answer was awarded 1 point, and incorrect or unsure responses were given 0 points. The overall knowledge score ranged from 0 to 34. Based on the total score, participants were categorized into three levels of knowledge: low knowledge (0–17), moderate knowledge (Greupner et al., 2020; El et al., 2016; AlMuammar et al., 2021; Zaidi et al., 2023; Shahid et al., 2022; AlMuammar et al., 2021; Simental-Mendía et al., 2016; von Luckner and Riederer, 2018; John et al., 2016; Alyami et al., 2020), and high knowledge (Landon and Young, 1993; De Oliveira et al., 2024; Rondanelli et al., 2021; Guerrera et al., 2009; Moretti, 2021; Knox et al., 2024; Launiala, 2009). Attitudes towards magnesium were measured using a Likert scale where participants responded to statements with values from 1 (strongly disagree) to 5 (strongly agree). The overall attitude score ranged from 1 to 5. Participants’ attitudes were classified as negative (1.0–2.49), neutral (2.5–3.49), or positive (3.5–5.0).

### Statistical analysis

Data were exported from Google^®^ Forms and analyzed using the Statistical Package for Social Sciences (SPSS^®^) version 24.0 (SPSS Inc., Chicago, IL, United States). Descriptive statistics were employed to analyze sociodemographic data. Continuous variables were summarized as means and standard deviations (SD), while categorical variables were presented as frequencies and percentages. The Shapiro-Wilk test was applied to assess the data normality. Questionnaire reliability was evaluated using Cronbach’s alpha, with a value of 0.8, indicating good internal consistency (values above 0.7 are considered acceptable).

Simple linear regression analysis was conducted to identify independent factors that might influence participants' knowledge and attitude regarding magnesium use. Variables with a p-value <0.25 in the univariate analysis were included in the multiple linear regression models. Multicollinearity between independent variables was tested by assessing tolerance values (>0.1) and Variance Inflation Factor (VIF) values (<10). No variable was excluded from the model as none showed multicollinearity. A p-value ≤0.05 was considered statistically significant for all analyses.

## Results

### Sociodemographic characteristics

A total of 1,470 responses were received in this study, 25 were excluded due to incompleteness. The study included 1,445 participants with a median (interquartile range (IQR)) age of 26 years (IQR ±60). The majority were female (62.8%) and single (54.3%). Participants were mainly from Jordan (30.3%), Egypt (27.2%), and Saudi Arabia (20.6%). Most participants (49.6%) had a background in medical specialties, and 57.8% reported having health insurance. A substantial proportion (45.0%) suffered from Vitamin D deficiency, and 21.5% reported having chronic diseases (Table 1).

**TABLE 1:** Sociodemographic characteristics of study participants (n = 1,445).

Variable	n	%
Gender		
• Female	907	62.8
• Male	538	37.2
Marital status		
• Single	784	54.3
• Married	607	42.0
• Others	54	3.7
Residencial country		
• Jordan	438	30.3
• Egypt	393	27.2
• Saudi Arabia	298	20.6
• Others	316	21.9
Monthly income of the family		
• Less than 300 dinars	388	26.9
• 300–700 dinars	438	30.3
• 701–1,000 dinars	279	19.3
• More than 1,000 dinars	340	23.5
The highest degree or level of education you have completed		
• Secondary education (high school diploma, Tawjihi, Baccalaureate)	120	8.3
• Vocational education	57	3.9
• Intermediate education (colleges, diploma)	202	14.0
• University education (Bachelor’s degree)	826	57.2
• Postgraduate studies (Master’s, PhD)	240	16.6
Occupation (profession)		
• Medical specialties (medicine, pharmacy, nursing, laboratories, etc.)	717	49.6
• Non-medical specialties (engineering, information technology, humanities, crafts, etc.)	267	18.5
• Student	207	14.3
• Retired	36	2.5
• Unemployed	196	13.6
• Other	22	1.5
Health insurance status		
• Insured	835	57.8
• Uninsured	610	42.2
If you have health insurance, does it cover dietary supplements?		
• Yes	140	9.7
• No	726	50.2
• Maybe	282	19.5
• Not applicable	297	20.6
Presence of chronic diseases		
Do you suffer from chronic diseases?		
• Yes	311	21.5
• No	1,134	78.5
If yes, what are these diseases?^a^		
• Diabetes	61	4.2
• Hypertension	94	6.5
• Cardiovascular disease	43	3.0
• Thyroid diseases	54	3.7
• Hyperlipidemia	105	7.3
• Obesity	128	8.9
• Sports injuries (such as slipped disc)	62	4.3
• Spinal column diseases (such as herniated disc)	108	7.5
• Others	78	5.4
Do you suffer from Vitamin D deficiency?		
• Yes	650	45.0
• No	410	28.4
• Unsure	385	26.6
Did you have sleeping problems in the last 6 months?		
• Always	179	12.4
• Often	352	24.4
• Sometimes	539	37.3
• Rarely	246	17.0
• Never	129	8.9
Do you have muscle problems such as frequent spasms or contractions?		
• Always	108	7.5
• Often	289	20.0
• Sometimes	510	35.3
• Rarely	324	22.4
• Never	214	14.8
Age (Years)	**Median ± IQR** **26 ± 60^b^**	

^a^ More than one answer was allowed.

^b^ Median (IQR).

### Knowledge of Arab communities regarding magnesium uses

In general, participants demonstrated low levels of knowledge about magnesium, with a mean knowledge score of 8.61 (±5.9) out of 34. As, the majority of participants had low knowledge (scoring 0–17). Table 2 showed that 76.5% correctly identified magnesium is not a treatment for high blood pressure, and 87.0% acknowledged the use magnesium for alleviating muscle spasms. Only 43.5% correctly recognized magnesium as a treatment for irregular heartbeat, and 21.2% knew it could be used for migraines. Side effects of magnesium such as fatigue (75.3%), headache (74.3%), and nausea and vomiting (38.8%), were commonly recognized.

**TABLE 2:** Participants’ responses for the knowledge items about magnesium (n = 1,445).

Question	Correct answer	
	n	%
Is it possible to use magnesium without a prescription?	664	46.0
Which of the following are indications for using magnesium?		
• Irregular heartbeat	629	43.5
• Constipation	547	37.9
• Respiratory diseases	213	14.7
• Migraine	306	21.2
• Preterm labor	208	14.4
• Preeclampsia	223	15.4
• Indigestion	438	30.3
• Maintaining bone health	784	54.3
• Improving blood sugar levels	388	26.9
• Treating high blood pressure	1,106	76.5
• Alleviating muscle spasms	1,257	87.0
Is it possible to use these medications with magnesium?		
• Panadol night	1,288	89.1
• Vitamin C	646	44.7
• Valium	152	10.5
• Muscle relaxants like Orphenadrine	1,084	75.0
• Valerian root	180	12.5
Is it possible to use magnesium as a preventive measure for muscle problems in the future?	1,215	84.1
Is it possible to use magnesium as a preventive measure for sleep disorders in the future?	1,200	83.0
What are the side effects of magnesium pills?		
• Nausea and vomiting	561	38.8
• Diarrhea	540	37.4
• Abdominal pain	472	32.7
• Headache	1,074	74.3
• Fatigue	1,088	75.3
• Weight gain	1,024	70.9
• Dark spots on the skin	1,087	75.2
• Depression	1,042	72.1
• Pancreatitis	1,073	74.3
• Urinary tract infection	1,095	75.8
• Blurred vision	176	12.2
• Dizziness and vertigo	301	20.8
• Fainting	191	13.2
• Other	160	11.1
• No side effects	936	64.8
Knowledge Score* (Mean, STD)	8.61, 5.9	

Low Knowledge: 0–17 (0%–50% of the total score), moderate knowledge: 18–27 (51%–80% of the total score), and high knowledge: 28–34 (81%–100% of the total score) (Krathwohl et al., 1964).

^*^ STD, standard deviation.

**FIGURE 1:**
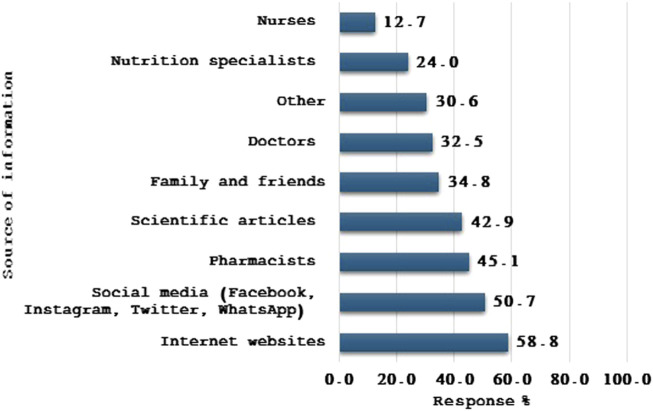
Source of information about magnesium used by the study participants (n = 1,445).

The primary sources of information about magnesium were the internet and websites (58.8%), followed by social media (50.6%) and pharmacists (45.1%) (Figure 1).

### Attitudes of Arab communities regarding magnesium uses

The overall attitude score was 3.02 (±0.66), indicating that most participants had a neutral attitude towards magnesium. Table 3 demonstrated that 43.6% agreed or strongly agreed that magnesium helps improve bone health, 34.8% believed that magnesium is effective for improving cardiovascular health, while 25.5% agreed it helps reduce migraines. Despite these attitudes, a significant number of participants (36.8%) were neutral regarding the safety of magnesium products.

**TABLE 3:** Participants’ responses for the attitude statements about magnesium (n = 1,445).

Statements	Strongly disagree	Disagree	Neutral	Agree	Strongly agree
	n (%)				
• Magnesium products are safe and can be used without fear of complications	139 (9.6)	371 (25.7)	532 (36.8)	309 (21.4)	94 (6.5)
• The effectiveness of magnesium products is guaranteed for their intended purposes	124 (8.6)	190 (13.1)	558 (36.6)	521 (36.1)	52 (3.6)
• The results of magnesium products have long-term benefits	93 (6.4)	279 (19.3)	823 (57.0)	223 (15.4)	27 (1.9)
• Magnesium products help improve bone health	140 (9.7)	174 (12.0)	471 (32.6)	543 (37.6)	117 (8.1)
• Magnesium products help control diabetes	122 (8.4)	239 (16.5)	815 (56.4)	225 (15.6)	44 (3.0)
• Magnesium products help improve cardiovascular health	125 (8.7)	180 (12.5)	638 (44.2)	426 (29.5)	76 (5.3)
• Magnesium products help reduce migraine episodes	93 (6.4)	181 (12.5)	802 (55.5)	317 (21.9)	52 (3.6)
• Magnesium products help reduce premenstrual symptoms	108 (7.5)	197 (13.6)	819 (56.7)	282 (19.5)	39 (2.7)
• Magnesium supplements help reduce anxiety attacks	113 (7.8)	150 (10.4)	718 (49.7)	404 (28.0)	60 (4.2)
Attitude Score* (Mean, STD)	3.02, 0.66				

Negative attitude scores between 1.0 and 2.49, neutral attitude (2.5–3.49), and positive attitude (3.5–5.0) (Likert, 1932).

^*^ STD, standard deviation.

### Practices of Arab communities regarding magnesium uses

In terms of practices, 59.9% of participants reported having used dietary supplements in the past 12 months, with 31.8% indicating they had used magnesium-containing products, Table 4. Among magnesium users, 36.1% had consulted a doctor before use, and only 23.7% had their magnesium levels checked prior to supplement use.

**TABLE 4:** Participants’ responses to the practice questions about magnesium (n= 1,445).

Question	n	%
Have you used any dietary supplements in the last 12 months?		
• No	579	40.1
• Yes	866	59.9
Have you used any products containing magnesium in the last 12 months?		
• No	985	68.2
• Yes	460	31.8
Have you consulted a doctor before using magnesium supplements?^a^		
• No	294	63.9
• Yes	166	36.1
Have you had your magnesium levels checked in your blood before using these supplements?^a^		
• No	351	76.3
• Yes	109	23.7
If you have used magnesium, what is the dosage (mg)?	Mean, STD	306.62, 200.5
If you have used magnesium, how many times per day do you take the dose?^a^		
• Once	372	80.9
• Twice	60	13.0
• Three times	28	6.1
On a scale from 1 (No benefit) to 10 (Benefited greatly), how would you rate the benefit of using magnesium?	Mean, STD	6.0, 2.6
Where do you usually purchase magnesium supplements?^a^		
• Pharmacy	425	92.4
• Doctor’s clinics	49	10.7
• Hospitals	71	15.4
• Facebook pages	37	8.0
• Family and friends abroad	55	12.0
• Commercial markets	46	10.0
• Other sources	52	11.3
What methods do you use to verify the source of magnesium supplements?^a^		
• Pharmacist’s Association label	274	59.6
• Barcode	161	35.0
• Manufacturer’s name	275	59.8
• Consulting with the pharmacist	316	68.7
• Other	105	22.8
Have you experienced any side effects when using magnesium supplements?^a^		
• No	387	84.1
• Yes	73	15.9
Do you regularly check medical information related to the supplements you use?^a^		
• Always	210	45.7
• Often	108	23.5
• Sometimes	103	22.4
• Rarely	30	6.5
• Never	9	2.0
Do you use any other medications or dietary supplements with magnesium?^a^		
• No	99	21.5
• Yes	361	78.5
If yes, what are these supplements?^a^		
• Vitamin D	333	72.4
• Omega 3	229	49.8
• Iron	195	42.4
• Folic Acid	144	31.3
• Other	180	39.1

^a^ These percentages were calculated based on the number of magnesium users in the study (n = 460).

The majority of users (80.9%) reported taking a magnesium supplement once per day, with an average dose of 306.62 mg (±200.5). A large proportion of users (92.4%) purchased their supplements from pharmacies, and 68.7% consulted with a pharmacist to verify the authenticity of the products they purchased.

Only 15.9% of participants reported experiencing side effects from magnesium supplements, with the most common side effects being nausea and diarrhea. Additionally, 45.7% regularly checked medical information related to the supplements they were using.

**FIGURE 2:**
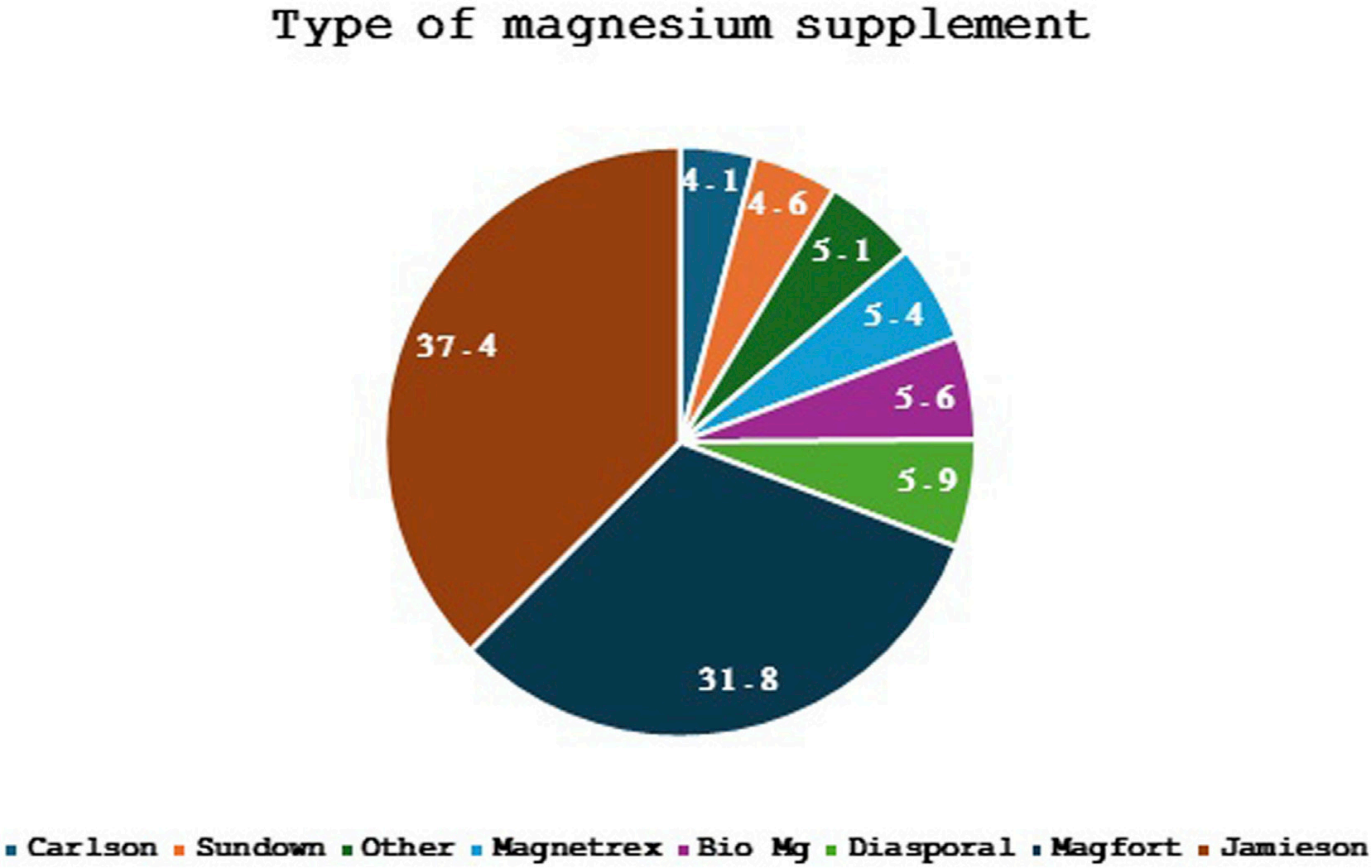
Types of magnesium supplement used by the participants (Only 390 responses were received for this question, presented data are the percentages).

In Figure 2, the types of magnesium supplements used by participants were illustrated. Among the 390 respondents who provided answers to this question, the most commonly used magnesium supplement brands were Magfort (Magnesium Bis-glycinate) (31.79%) and Diasporal (magnesium citrate) (37.43%), followed by other popular brands like Jamieson (Magnesium (oxide, citrate, fumarate, malate and succinate)) (5.89%), Bio Mg (Magnesium Bis-glycinate) (5.38%), and Magnetrex (magnesium, vitamin B6, Citrus sinensis and taurine) (5.12%). Lesser-used brands included Carlson (Magnesium glycinate) (4.10%) and Sundown (Magnesium oxide) (4.61%).

### Factors affecting the participants’ knowledge and attitude toward magnesium

A linear regression analysis was conducted to assess the factors affecting both knowledge and attitude. Several factors were found to be significantly associated with the knowledge score, while others showed significance in predicting attitude. For knowledge, participants with polycystic ovary syndrome had significantly higher knowledge scores compared to those without the condition (β = 0.175, p = 0.033). Information sources also played a critical role in enhancing knowledge. Specifically, obtaining information from pharmacists (β = 0.188, p = 0.019), scientific articles (β = 0.247, p = 0.004), and the internet or websites (β = 0.182, p = 0.048) were all significant positive factor of higher knowledge scores. Additionally, participants who reported experiencing side effects from magnesium supplements demonstrated a significantly higher knowledge score (β = 0.281, p = 0.001), suggesting that personal health experiences may increase awareness and knowledge.

Regarding attitude, while no factors reached statistical significance at the conventional threshold (p < 0.05), certain variables showed a trend toward significance. These included country of residence (β = 0.181, p = 0.077), obesity (β = 0.205, p = 0.083), and obtaining information from nutritionists (β = −0.189, p = 0.075). Furthermore, participants who had used dietary supplements in the past 12 months showed a marginally significant association with a more positive attitude (β = 0.182, p = 0.073). Knowledge score itself also had a borderline positive association with attitude (β = 0.216, p = 0.054) (Table 5).

**TABLE 5:** Linear regression analysis of factors affecting the knowledge and attitude toward magnesium.

Variable	Knowledge		Attitude	
	Beta	p-value	Beta	p-value
• Age (years)	0.166	0.164	—	—
• Marital status	−0.166	0.115	—	—
• Do you have health insurance?	−0.112	0.153	—	—
• Do you suffer from Vitamin D deficiency?	0.12	0.146	—	—
• Polycystic ovary syndrome	0.175	0.033*	−0.079	0.431
• Sleeping problems (last 6 months)	−0.153	0.073	—	—
• Information source (Pharmacist)	0.188	0.019*	—	—
• Information source (Scientific articles)	0.247	0.004*	—	—
• Information source (Internet and websites)	0.182	0.048*	−0.061	0.583
• Attitude	0.149	0.054	—	—
• Experienced side effects from magnesium supplements	0.281	0.001*	—	—
• Country of residence	—	—	0.181	0.077
• Health insurance covers dietary supplements	—	—	0.142	0.161
• Obesity	—	—	0.205	0.083
• Information source (Physician)	—	—	0.18	0.093
• Information source (Nutritionist)	—	—	−0.189	0.075
• Used dietary supplements (last 12 months)	—	—	0.182	0.073
• Knowledge score	—	—	0.216	0.054

*p < 0.05 indicates statistical significance.

## Discussion

Public awareness regarding dietary supplements varies across different regions and demographics (AlTamimi, 2019). However, there remains lack of evidence about public awareness regarding magnesium use in the Arab regions. Considering the importance of exploring the underlying factors determining knowledge, attitudes, and practices related to magnesium supplements across different cultural contexts. This study was the first multinational cross-sectional study to explore the knowledge, attitudes, and practices of public in Arab countries regarding magnesium supplements.

The results of the present study revealed that participants demonstrated a low total knowledge score about magnesium, with a mean score of 8.61 ± 5.9 out of 34, indicating that participants have a limited understanding of magnesium-related information. The overall attitude score was 3.02 (±0.66), indicating that most participants had a neutral attitude towards magnesium. In terms of practices, 59.9% of participants reported using dietary supplements in the past 12 months, with 31.8% indicating they had used magnesium-containing products. The knowledge gab might be illustrated by the fact that magnesium supplements are widely self-prescribed as an over-the-counter (OTC) product. In-contrast, prescribed medicines are typically provided through healthcare providers who offer detailed information about the drug, including its indications, dosage, potential adverse drug reactions, and interactions (Khan et al., 2023). This is the first study to evaluate the public knowledge about magnesium supplements. Nevertheless, these findings are consistent with those of Alqrache et al., who assessed the patterns of dietary supplement use among students in Saudi Arabia, revealing a similar lack of awareness about their roles in health and disease (Alqrache et al., 2021). Similarly, Aghamohammadi et al. found significant deficiencies in nutritional knowledge among medical students in Iran, further highlighting gaps in understanding key dietary components (Dolatkhah et al., 2019). The findings are also consistent with the general lack of knowledge reported among Arab populations regarding nutritional supplement knowledge (Alahmadi and Albassam, 2023; Sharma et al., 2014; Wilson et al., 2010; Mullan et al., 2017; Alahmadi and Albassam, 2023). Despite the low overall knowledge score reported among the studied sample, the magnesium’s role in alleviating muscle spasms, as a preventive agent for muscle-related problems, and sleep disorders were highly recognized (87%, 84.1%, and 83%, respectively). This suggests that public is generally well-informed about magnesium role in these specific areas. This awareness may be due to widespread information on magnesium’s muscle-relaxing properties and its use in sports and general wellness (Souza et al., 2023). However, the study also identified a general lack of awareness regarding magnesium’s broader health benefits and its applications in some medical conditions, such as preterm labor, respiratory diseases, and preeclampsia.

In terms of knowledge about magnesium-related side effects, the recognition of side effects showed varied patterns for different side effects associated with magnesium use. While some side effects like urinary tract infections and fatigue were widely acknowledged, others such as blurred vision and fainting were less recognized. This pattern perhaps resulted from different sources of information or personal experiences (O’Donovan et al., 2019). On the contrary, the results of the regression analysis which indicated that presence of certain health problems, such as polycystic ovarian syndrome, notably was associated with increased knowledge levels.

Our study demonstrated that the primary sources of information about magnesium were the internet websites (58.8%), followed by social media (50.6%) and pharmacists (45.1%). The primary source of information was found to be a significant determinant of participants’ level of knowledge. For instance, obtaining information from pharmacists, scientific articles, and the internet/websites were all significant positive factors associated with higher knowledge scores. Generally, sources of information on dietary supplements varied greatly among previous studies in Arab regions (Mukattash et al., 2022; Alyami et al., 2020; Amer et al., 2024; Alsofyani et al., 2018). Amer et al. (2024), demonstrated that the main sources of information about dietary supplements were healthcare providers, with almost 80%, and specialized coaches, with 78.3%. Nonetheless, Alsofyani et al. reported that magazines, scientific books, and the internet were the three main sources of information (Alsofyani et al., 2018).

Collectively, the low levels of knowledge reported in the present study highlights the urgent need to plan targeted educational initiatives to enhance the public awareness about nutritional supplements. Such initiatives have been previously linked to improving public awareness about different drugs (Thong et al., 2021; Shen et al., 2021), and health conditions (Almutairi et al., 2023; Afzal et al., 2023). In this regard, pharmacists play a crucial role (Alkhawajah and Eferakeya, 1992), particularly for the areas with the low awareness levels such as magnesium use in conditions with significant knowledge gap, such as its use in obstetrics conditions and respiratory diseases.

In terms of attitude, the results of the present study revealed varying levels of confidence in the safety, and the effectiveness of magnesium supplements across different health areas. The overall attitude score was 3.02 ± 0.66, indicating neutral attitude. The areas with highest neutral responses were premenstrual symptoms 56.7% diabetes control 56.4%, and migraine episodes 55.5%. Conversely, neutral beliefs about the benefits of magnesium for bone health were only reported by 32.6% of the studied sample, indicating higher confidence levels in these areas specifically. This might be justified by the high knowledge scores in these areas. In line with this explanation the results of the regression analysis indicating that knowledge score had a positive association with attitude. These findings highlight the importance of addressing areas of uncertainty with clear evidence-based information.

In terms of practice, the substantial gap between general supplement uses 59.9% and magnesium-specific use 31.8% suggests that, while supplements are popular, magnesium is less commonly used, indicating lack of perceived necessity to magnesium. A similar pattern was recently reported in a multinational cross-sectional study in six Arab countries (Amer et al., 2024). Despite the prevalent use of vitamin C, vitamin D, iron, and zinc, other mineral supplements like magnesium and selenium were rarely utilized in significant proportion (66.8, and 84.4%, respectively) of the studied sample (Amer et al., 2024).

A concerning finding also was the low rate of pre-usage medical consultation 36.1%, and blood level testing 23.7%. The use of supplements without medical supervision, reported in the present study, reflects the globally prevalent irrational use of medicines (Shankar, 2009; Mao et al., 2015), including the Arab countries (Mhadi et al., 2023). This lack of medical oversight indicates potential risks of inappropriate supplementation, which could lead to adverse health outcomes. Another notable observation was the high rate of co-supplementation 78.5% with other products like Vitamin D, which raises potential interaction risks. For example, use of magnesium-containing products with a vitamin D analog may increase the risk of hypermagnesemia, particularly in chronic renal dialysis patients (Drugs.com, 2024).

The present study is the first multinational study to assess Arab communities’ knowledge, attitudes and practices towards magnesium supplements. The study is strengthened by its multinational study design, and focus on magnesium supplement use specifically, along with its influential factors. The results of this study might help the health authorities to plan educational initiatives to enhance public awareness about magnesium use. Nonetheless, some limitations should be considered when interpreting the results of this study. The first notable limitation, is the small sample size. The observational study design which introduces risk of bias that may affect the reliability and validity of the findings: the use of online survey tool may introduce selection bias, and recall bias may also exist. The data used in the analysis of this study were self-reported, which might introduce reporting bias. Considering the present study surveyed participants from Arab countries only, limiting the generalizability of the findings to other cultural contexts. Thus, further similar studies are warranted in a diverse range of countries, encompassing other populations.

## Conclusion

The study revealed a significant knowledge gap regarding magnesium, with participants demonstrating limited awareness of its broader health benefits and medical applications. Additionally, the substantial gap between general supplement use and magnesium-specific use suggests a lack of perceived necessity for magnesium. Our findings suggest that Arab communities also have neutral attitude towards magnesium supplements. The results of this study suggest that more emphasis should be placed on targeted educational initiatives to enhance the public awareness about nutritional supplements, particularly magnesium. The findings may help policymakers identify the knowledge gap, and the areas of uncertainty about magnesium with clear evidence-based information. Further larger studies are warranted in a diverse range of countries, are needed to confirm this finding.

## Data Availability

The original contributions presented in the study are included in the article/Supplementary Material, further inquiries can be directed to the corresponding author.
