# Supplementary material for: A multinational cross-sectional study on knowledge, attitudes, and practices towards magnesium supplements
Source: Front Pharmacol. 2025 Apr 28;16:1550695. doi: 10.3389/fphar.2025.1550695 (PMC12066956; doi:10.3389/fphar.2025.1550695)
Supplement: Supplementary file 1 [file Supplementaryfile1.docx]

**Assessment of Arab Communities Knowledge, Attitude and Practices on the Uses and Misuses of Magnesium Supplements: A multinational Study**

Section 1

A group of Arab researchers is studying the extent of knowledge, awareness, and practices of Arab communities regarding the uses of magnesium, which are used as dietary supplements. A group of researchers from Al-Zarqa University and Applied Science Private University is studying the extent of knowledge, attitude, and practices of the Arab community regarding the uses of magnesium, which are used as dietary supplements.

Your participation in filling out this questionnaire is voluntary and is appreciated. Please note that the information provided will remain confidential and will only be used for research purposes. The identity of the participants will be kept confidential, and you can withdraw from participation at any time during the questionnaire.

For further information contact the principal researcher via the following email: [rabutaima@zu.edu.jo](mailto:rabutaima@zu.edu.jo)

Your participation in filling out this questionnaire is voluntary and will be treated confidentially. Would you like to participate?

Yes

No

Section 2

**Personal Information**

**Gender:**

Male 1

Female 2

**Age (in years)**: ......................

**Marital Status:**

Single 1

Married 2

Other 3

**Place of Residence:**

Jordan 1

Egypt 2

Saudi Arabia 3

Others 4

**Monthly Family Income (in US dollars):**

Less than 300 USD 1

300-700 USD 2

701-1000 USD 3

More than 1000 USD 4

**Education:**

Secondary education (high school diploma, Tawjihi, Baccalaureate) 1

Intermediate education (colleges, diploma) 2

Vocational education 3

University education (Bachelor's degree) 4

Postgraduate studies (Master's, PhD) 5

**Field of Work:**

Medical specialties (medicine, pharmacy, nursing, laboratories, etc.) 1

Non-medical specialties (engineering, information technology, humanities, crafts, etc.)2

Unemployed 3

Student 4

Retired 5

**Do you have health insurance?**

Yes 1

No 0

**If you have health insurance, does it cover dietary supplements?**

Yes 1

No 0

Maybe 0

Not applicable 0

**Do you suffer from Vitamin D deficiency?**

Yes 1

No 0

Unsure 0

**Do you suffer from any chronic diseases such as (hypertension, diabetes, etc.)?**

Yes 1

No 0

**If yes, what are these diseases?**

Diabetes: Yes / No 1/0

Hypertension: Yes / No

Cardiovascular Diseases: Yes / No

Thyroid diseases: Yes / No

hypercholesterolemia and Hyperlipidemia: Yes / No

Obesity: Yes / No

Respiratory diseases: Yes / No

Sports injuries (such as slipped disc): Yes / No

Spinal column diseases (such as herniated disc)*:* Yes / No

Psychological diseases (depression, bipolar disorder, schizophrenia): Yes / No

Endometriosis: Yes / No

Polycystic ovary syndrome: Yes / No

Other: Yes / No

**Did you have sleeping problems in the last six months?**

Always 5

Often 4

Sometimes 3

Rarely 2

Never 1

**Do you have muscle problems such as frequent spasms or contractions?**

Always 5

Often 4

Sometimes 3

Rarely 2

Never 1

Section 3

**Knowledge of Arab Communities Regarding Magnesium Uses**

**Based on your knowledge, is it possible to use magnesium without a prescription?**

| **Answer** | **Score (False: 0, True: 1)** |
| --- | --- |
| **Yes** | **1** |
| **No** | **0** |
| **Not sure** | **0** |

**Based on your knowledge, which of the following are reasons for using magnesium? Score (False: 0, True: 1, Not sure: 0))**

| **Answer** | **Yes** | **No** | **Not sure** |
| --- | --- | --- | --- |
| **Irregular heartbeat** | **1** | **0** | **0** |
| **Constipation** | **1** | **0** | **0** |
| **Respiratory diseases** | **1** | **0** | **0** |
| **Migraine** | **1** | **0** | **0** |
| **Preterm labor** | **1** | **0** |  |
| **Preeclampsia** | **1** | **0** | **0** |
| **Indigestion** | **1** | **0** | **0** |
| **Maintaining bone health** | **1** | **0** | **0** |
| **Improving blood sugar levels** | **1** | **0** | **0** |
| **Treating high blood pressure** | **0** | **1** | **0** |
| **Alleviating muscle spasms** | **0** | **1** | **0** |

**Based on your knowledge, is it possible to use these medications with magnesium? Score (False: 0, True: 1, Not sure: 0))**

| **Answer** | **Yes** | **No** | **Not sure** |
| --- | --- | --- | --- |
| **Panadol night** | **0** | **1** | **0** |
| **Vitamin C** | **1** | **0** | **0** |
| **Valium** | **1** | **0** | **0** |
| **Muscle relaxants like Orphenadrine** | **0** | **1** | **0** |
| **Valerian root** | **1** | **0** | **0** |

**Based on your knowledge, is it possible to use magnesium as a preventive measure for muscle problems in the future?**

| **Answer** | **Score (False: 0, True: 1)** |
| --- | --- |
| **Yes** | **0** |
| **No** | **1** |
| **Not sure** | **0** |

**Based on your knowledge, is it possible to use magnesium as a preventive measure for sleep disorders in the future?**

| **Answer** |  | **Score (False: 0, True: 1)** |
| --- | --- | --- |
| **Yes** |  | **0** |
| **No** |  | **1** |
| **Not sure** |  | **0** |

**Based on your knowledge, what are the side effects of magnesium pills? Score (False: 0, True: 1, Not sure: 0))**

| **Answer** | **Yes** | **No** | **Not sure** |
| --- | --- | --- | --- |
| **Nausea and vomiting** | **1** | **0** | **0** |
| **Diarrhea** | **1** | **0** | **0** |
| **Abdominal pain** | **1** | **0** | **0** |
| **Headache** | **0** | **1** | **0** |
| **Fatigue** | **0** | **1** | **0** |
| **Weight gain** | **0** | **1** | **0** |
| **Dark spots on the skin** | **0** | **1** | **0** |
| **Depression** | **0** | **1** | **0** |
| **Pancreatitis** | **0** | **1** | **0** |
| **Urinary tract infection** | **0** | **1** | **0** |
| **Blurred vision** | **1** | **0** | **0** |
| **Dizziness and vertigo** | **1** | **0** | **0** |
| **Fainting** | **1** | **0** | **0** |
| **Other** | **1** | **0** | **0** |
| **No side effects** | **0** | **1** | **0** |

**What is the source of your information about the effectiveness of this dietary mineral? (yes/no, 1/0)**

Doctors

Pharmacists

Nurses

Nutrition specialists

Social media (Facebook, Instagram, Twitter, WhatsApp)

Scientific articles

Family and friends

Internet websites

Other

Section 4

**Awareness of Arab Communities Regarding Magnesium Uses**

**Choose your level of agreement with the following statements regarding the use of magnesium products:**

**Strongly agree / Agree / Neutral / Disagree / Strongly disagree (5/4/3/2/1)**

Magnesium products are safe and can be used without fear of complications

The effectiveness of magnesium products is guaranteed for their intended purposes

The results of magnesium products have long-term benefits

Magnesium products help improve bone health

Magnesium products help control diabetes

Magnesium products help improve cardiovascular health

Magnesium products help reduce migraine episodes

Magnesium products help reduce premenstrual symptoms

Magnesium supplements help reduce anxiety attacks

Section 5

**Practices of Arab Communities Regarding the Use of Magnesium**

**Have you used any dietary supplements in the last 12 months?**

Yes 1

No 0

**Have you used any products containing magnesium in the last 12 months?**

Yes 1

No 0

Section 6

**Practices of Arab Communities Regarding the Use of Magnesium**

**Have you consulted a doctor before using magnesium supplements?**

Yes 1

No 0

**Have you had your magnesium levels checked in your blood before using these supplements?**

Yes 1

No 0

**If you have used magnesium, what is the dosage (mg)?** ............................

**If you have used magnesium, how many times per day do you take the dose?** ....................................................

**On a scale from 1 to 10, how would you rate the benefit of using magnesium?**

Benefited greatly Did not benefit at all

10 9 8 7 6 5 4 3 2 1

**What is the name or type of magnesium supplement you have used?**

Jamieson

Diasporal

Sundown

Carlson

Bio Mg

Magfort

Magnetrex

Other

**Where do you usually purchase magnesium supplements?**

Pharmacy Yes / No

Doctor's clinics Yes / No

Hospitals Yes / No

Facebook pages Yes / No

Family and friends abroad Yes / No

Commercial markets Yes / No

Other sources Yes / No

**What methods do you use to verify the source of magnesium supplements?**

Pharmacist's Association label Yes / No

Barcode Yes / No

Manufacturer's name Yes / No

Consulting with the pharmacist Yes / No

Other Yes / No

**Have you experienced any side effects when using magnesium supplements?**

Yes 1

No 0

**Do you regularly check medical information related to the supplements you use?**

Always 5

Often 4

Sometimes 3

Rarely 2

Never 1

**Do you use any other medications or dietary supplements with magnesium?**

Yes 1

No 0

**If yes, what are these supplements?**

Vitamin D

Omega 3

Iron

Folic Acid

Other
